# *Ccn2* Deletion Reduces Cardiac Dysfunction, Oxidative Markers, and Fibrosis Induced by Doxorubicin Administration in Mice

**DOI:** 10.3390/ijms25179617

**Published:** 2024-09-05

**Authors:** Antonio Tejera-Muñoz, Marcelino Cortés, Alianet Rodriguez-Rodriguez, Lucia Tejedor-Santamaria, Vanessa Marchant, Sandra Rayego-Mateos, Maria José Gimeno-Longas, Andrew Leask, Tri Q. Nguyen, María Martín, Jose Tuñón, Isabel Rodríguez, Marta Ruiz-Ortega, Raul R. Rodrigues-Díez

**Affiliations:** 1 Research Unit, Complejo Hospitalario La Mancha Centro, 13600 Alcázar de San Juan, Spain; antoniotemu@gmail.com; 2 Instituto de Investigación Sanitaria de Castilla-La Mancha (IDISCAM), 45004 Toledo, Spain; 3 Cardiology Department, Hospital Universitario Fundación Jiménez Díaz, 28040 Madrid, Spain; mcortesg@quironsalud.es (M.C.); jtunon@quironsalud.es (J.T.); 4 Departamento de Biología de Sistemas, Universidad de Alcalá, 28871 Alcalá de Henares, Spain; alianet206@gmail.com; 5 Cellular and Molecular Biology in Renal and Vascular Pathology Laboratory, Instituto de Investigación Sanitaria-Fundación Jiménez Díaz, Universidad Autónoma de Madrid, 28040 Madrid, Spain; lucia.tejedor@quironsalud.es (L.T.-S.); vmarchant.hernandez@gmail.com (V.M.); srayego@quironsalud.es (S.R.-M.); 6 RICORS2040, Instituto de Salud Carlos III, 28040 Madrid, Spain; 7 Department of Cell Biology, School of Medicine, Universidad Complutense de Madrid, 28040 Madrid, Spain; margim08@ucm.es; 8 College of Dentistry, University of Saskatchewan, 105 Wiggins Road, Saskatoon, SK S7N 5E4, Canada; anl312@mail.usask.ca; 9 Department of Pathology, University Medical Center Utrecht, Heidelberglaan 100, 3584 CX Utrecht, The Netherlands; t.q.nguyen@umcutrecht.nl; 10 Cardiology Department, Hospital Universitario Central de Asturias, 33011 Oviedo, Spain; mmartinf7@hotmail.com; 11 Cardiac Pathology Research Group, Instituto de Investigación Sanitaria del Principado de Asturias (ISPA), 33011 Oviedo, Spain; isabel.rodriguez@ispasturias.es

**Keywords:** CCN2, doxorubicin, cardiac dysfunction, oxidative stress, fibrosis

## Abstract

Cellular Communication Network Factor 2 (CCN2) is a matricellular protein implicated in cell communication and microenvironmental signaling. Overexpression of CCN2 has been documented in various cardiovascular pathologies, wherein it may exert either deleterious or protective effects depending on the pathological context, thereby suggesting that its role in the cardiovascular system is not yet fully elucidated. In this study, we aimed to investigate the effects of *Ccn2* gene deletion on the progression of acute cardiac injury induced by doxorubicin (DOX), a widely utilized chemotherapeutic agent. To this end, we employed conditional knockout (KO) mice for the *Ccn2* gene (CCN2-KO), which were administered DOX and compared to DOX-treated wild-type (WT) control mice. Our findings demonstrated that the ablation of CCN2 ameliorated DOX-induced cardiac dysfunction, as evidenced by improvements in ejection fraction (EF) and fractional shortening (FS) of the left ventricle. Furthermore, DOX-treated CCN2-KO mice exhibited a significant reduction in the gene expression and activation of oxidative stress markers (Hmox1 and Nfe2l2/NRF2) relative to DOX-treated WT controls. Additionally, the deletion of *Ccn2* markedly attenuated DOX-induced cardiac fibrosis. Collectively, these results suggest that CCN2 plays a pivotal role in the pathogenesis of DOX-mediated cardiotoxicity by modulating oxidative stress and fibrotic pathways. These findings provide a novel avenue for future investigations to explore the therapeutic potential of targeting CCN2 in the prevention of DOX-induced cardiac dysfunction.

## 1. Introduction

Cellular Communication Network Factor 2 (CCN2), also known as Connective Tissue Growth Factor (CTGF), is an extracellular matrix (ECM) protein involved in cellular communication and microenvironmental signaling responses [1,2]. CCN2 belongs to the CCN protein family, which includes five other members that share a conserved tetramodular structure [3]. This structure consists of four functional modules and a central hinge region that is susceptible to cleavage by proteases, resulting in two fragments: the N-terminal and C-terminal [1,2]. Even though each module can individually perform different biological functions due to its multiple binding sites, the C-terminal segment exhibits similar effects as the entire CCN2 molecule. Consequently, it has been employed in fundamental and preclinical research to investigate its role in both normal and disease conditions [4].

CCN2 performs a wide variety of context-dependent biological roles, such as regulating cell growth, differentiation, development, adhesion, inflammation, and ECM remodeling [1,2,5]. In the cardiovascular system, CCN2 is highly expressed during heart and blood vessel development and is overexpressed in several diseases [6,7,8]. CCN2 is significantly involved in fibrogenesis and is a well-recognized biomarker for fibrosis, acting as a key downstream mediator of TGF-β (transforming growth factor-beta) and Ang II (angiotensin II) [9,10,11]. Based on these studies, CCN2 was initially proposed as a growth factor and cytokine. Still, recent findings have expanded its role to include functioning as a matricellular ECM protein involved in cell communication and the coordination of responses to microenvironmental signals [12,13,14].

Initially, CCN2 was believed to be absent in healthy adult tissues, with its overexpression linked exclusively to pathological conditions and injury responses. Recent evidence indicates that CCN2 is constitutively expressed in specific tissues, particularly in vascular smooth muscle [5,15,16]. CCN2 has been implicated in various cardiovascular conditions, including heart failure, pulmonary hypertension, restenosis, and atherosclerosis, both in experimental models and in clinical settings [7]. In this context, it has been proposed as a biomarker for cardiac dysfunction in chronic heart failure and myocardial fibrosis [17]. Experimental studies have shown that blocking CCN2 is beneficial for pulmonary vascular remodeling and heart failure [18,19]. Conversely, overexpression of CCN2 in cardiomyocytes has been shown to provide protection against cardiac pressure overload, ischemia–reperfusion injury, and stroke [20,21,22]. Moreover, recombinant CCN2 has been found to reduce infarct size and improve cardiac function following ischemia [23]. Interestingly, the absence of CCN2 increases susceptibility to aortic aneurysms in angiotensin II-induced hypertension [5] and worsens experimental atherosclerosis [24]. These findings highlight the crucial role of CCN2 in maintaining cardiovascular homeostasis and demonstrate its variable effects across different cardiovascular pathologies, warranting further investigation.

Doxorubicin (DOX; also called Adriamycin) is an anthracycline anticancer drug widely used in chemotherapy for treating various types of cancer. Despite its therapeutic efficacy, DOX is associated with significant cardiotoxicity, which limits its clinical application [25]. Recent studies demonstrated that DOX-induced cardiac side effects are mediated by the modulation of several cellular responses, including oxidative stress, apoptosis, and fibrosis [26,27]. Understanding the mechanisms underlying DOX-induced cardiotoxicity is crucial for developing strategies to mitigate its adverse cardiac effects and improve the therapeutic index of this important chemotherapeutic agent. In this sense, recent in vitro studies demonstrated that downregulation of CCN2 expression reduced cardiomyocyte injury after DOX administration [28]. Thus, considering this data and the above-mentioned background about the role of CCN2 in a wide range of cellular responses, the present study aims to investigate the effects of *Ccn2* gene deletion on the progression of DOX-induced cardiac damage in mice.

## 2. Results

### 2.1. Absence of CCN2 Does Not Alter DOX-Induced Body Weight and Cardiac Mass Loss in Mice

**Figure 1:**
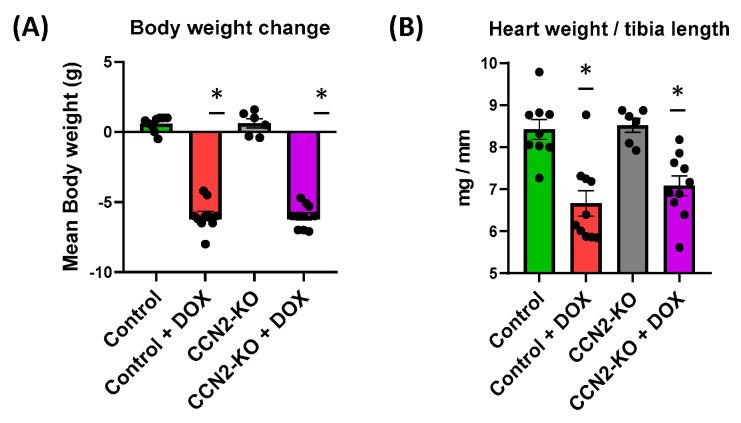
Effects of DOX administration on body weight and cardiac mass in mice. Effects of DOX administration in heart weight (**A**) and the ratio of heart weight to tibia length (**B**) in mice. * *p* < 0.05 vs. Control. *n* = 6–10.

Mice in both DOX-treated groups (Control + DOX and CCN2-KO + DOX) exhibited significant weight loss compared to the untreated groups (Control and CCN2-KO) (Figure 1A). Additionally, DOX administration resulted in a significant decrease in the heart-weight-to-tibia-length ratio compared to untreated mice. In CCN2-KO mice, DOX administration did not result in significant differences in cardiac mass loss compared to the Control + DOX group (Figure 1B). These findings indicate that the absence of CCN2 does not influence the weight or cardiac mass loss induced by DOX administration. All these data demonstrate that the deletion of Ccn2 does not influence either the body weight or the cardiac mass loss induced by DOX administration.

### 2.2. Deletion of Ccn2 in Mice Reduces DOX-Induced Cardiac Dysfunction

**Figure 2:**
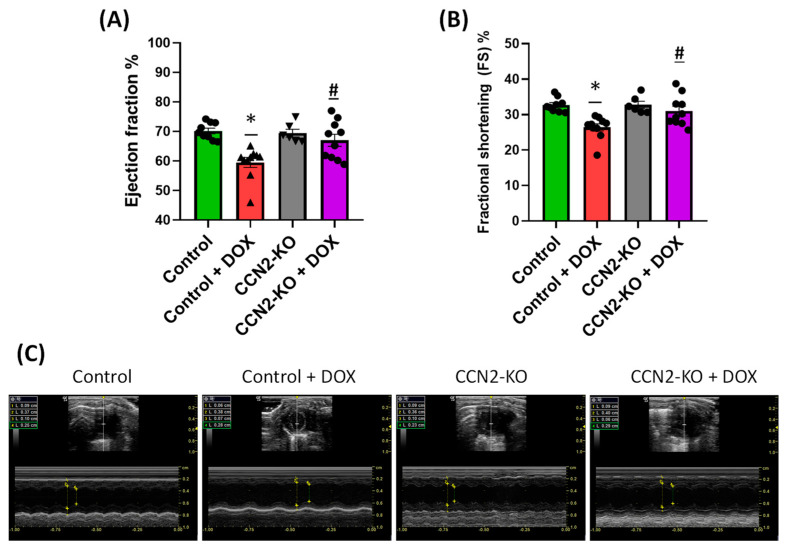
Impact of DOX administration on cardiac function in mice. Changes in values of ejection fraction (EF) (**A**) and fractional shortening (**B**) (expressed as percentages) with and without DOX administration in mice. (**C**) M-mode echocardiography representative images obtained five days post-DOX administration for the assessment of cardiac function (See Supplementary Figure S1 for enlarged images). * *p* < 0.05 vs. Control; # *p* < 0.05 vs. Control + DOX. *n* = 6–10.

The impact of DOX on cardiac function in mice was assessed five days post-administration using M-mode echocardiography in each mouse. Images, as depicted in Figure 2A, were obtained for measurement, enabling the assessment of ejection fraction (EF) and fractional shortening (FS). Measurements of EF and FS values were calculated based on the collected data (Supplementary Figure S1). As shown in the graphs, DOX treatment led to a significant decrease in both EF and FS in control mice (Control + DOX group) compared to the other experimental groups (Figure 2B). In contrast, DOX administration in CCN2-KO mice (CCN2-KO + DOX group) did not result in significant changes in EF and FS values compared to the untreated groups (Control and CCN2-KO groups) (Figure 2C and Supplementary Figure S2). These results suggest that CCN2 could participate in cardiac dysfunction induced by DOX.

### 2.3. CCN2 Deletion Modulates the Effects of DOX on Gene Expression Markers of Oxidative Stress and Cardiac Damage in Mouse Heart

**Figure 3:**
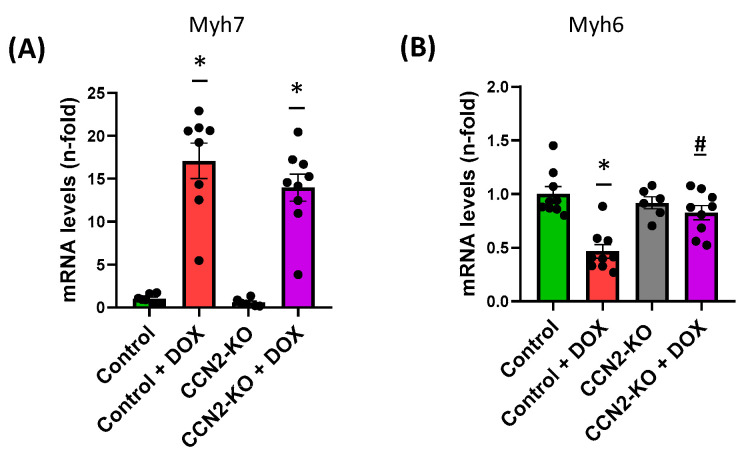
Gene expression related to cardiac damage in the hearts of study mice. The expression levels of *Myh7* (**A**) in the different experimental groups show a significant increase in both the Control + DOX and CCN2-KO + DOX groups compared to the groups without DOX treatment. For *Myh6* (**B**), DOX administration has varying effects depending on the deletion of CCN2, resulting in a significant decrease in the Control group, while this change is not observed in the CCN2-KO group. * *p* < 0.05 vs. Control; # *p* < 0.05 vs. Control + DOX. *n* = 6–10.

Gene expression related to cardiac damage, apoptosis, and oxidative stress in the hearts of the studied mice was analyzed using RT-qPCR from samples collected 5 days post-DOX administration. Regarding genes associated with cardiac damage, DOX induced a significant increase in *Myh7* expression in both Control + DOX and CCN2-KO + DOX groups compared to mice in groups without DOX treatment (Figure 3A). However, the expression of *Myh6* decreased in the control + DOX group compared to the control group, while in the CCN2-KO + DOX group, no significant differences were detected compared to other experimental groups (Figure 3B).

**Figure 4:**
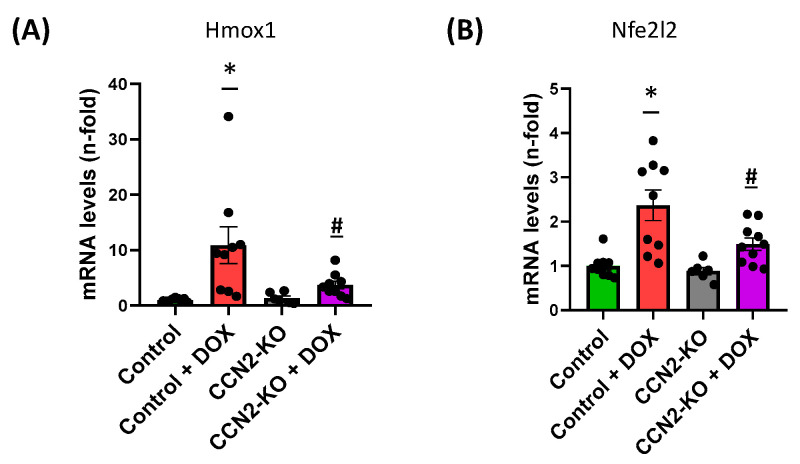
Gene expression related to oxidative stress in the hearts of studied mice. The expression levels of *Hmox1* (**A**) and *Nfe2l2* (**B**) in the different experimental groups show a significant increase in the Control + DOX group compared to the Control group without DOX treatment. Additionally, for both genes, DOX administration in the absence of CCN2 results in a significant reduction in the increase compared to the Control + DOX group. * *p* < 0.05 vs. Control; # *p* < 0.05 vs. Control + DOX. *n* = 6–10.

Furthermore, measuring the expression of genes involved in oxidative stress revealed a significant increase in *Hmox1* and *Nfe2l2* expression in the control + DOX group compared to other trial groups (Figure 4A,B). Conversely, the absence of CCN2 reduced the increase in *Hmox1* and *Nfe2l2* levels following DOX administration compared to the control + DOX group, showing no variations compared to the Control group.

**Figure 5:**
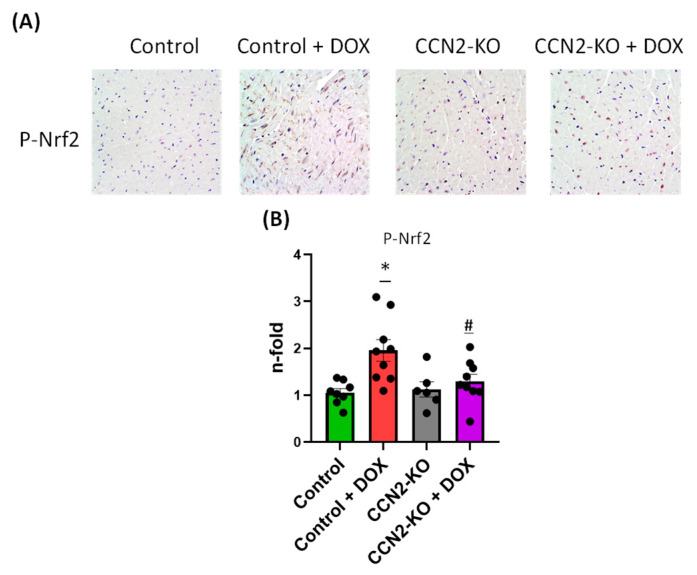
Immunohistochemistry (IHC) detection of NRF2 protein activation in the left ventricular tissue of mouse hearts. (**A**) Representative IHC images showing phospho-NRF2 staining. (**B**) Quantification of positive staining for phospho-NRF2 (p-NRF2) levels. Five days of DOX administration resulted in significantly elevated phospho-NRF2 levels in the Control group mice. In contrast, the CCN2-KO + DOX group did not show significant changes in protein activation compared to their corresponding CCN2-KO control group. * *p* < 0.05 vs. Control; # *p* < 0.05 vs. Control + DOX. *n* = 6–10.

Immunohistochemistry (IHC) was used to detect the activation of Nrf2 protein (phospho-Nrf2; codified by *Nfe2l2* gene) in the left ventricular tissue of mouse hearts, as shown in Figure 5. Analysis of positive staining revealed elevated phospho-NRF2 levels (p-NRF2) after five days of DOX administration in the Control group mice. In contrast, the CCN2-KO + DOX group displayed no significant changes in protein activation compared to their corresponding CCN2-KO control group (Figure 5). These results suggest CCN2 might play a role in regulating oxidative stress in cardiac tissue.

**Figure 6:**
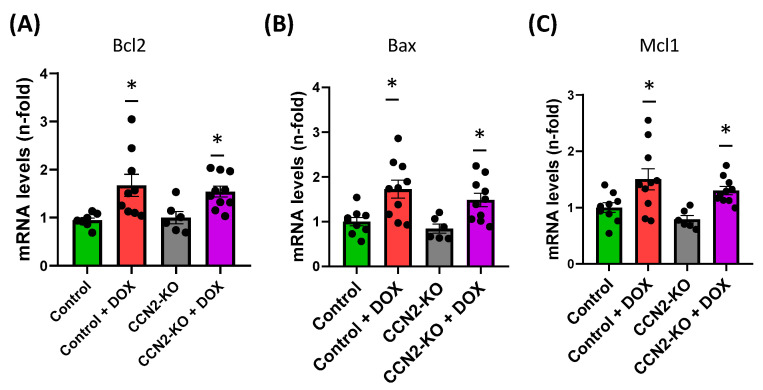
DOX administration increases apoptosis biomarkers in mouse hearts. (**A**) *Bcl2*, (**B**) *Bax*, and (**C**) *Mcl1* levels were measured, showing a significant increase in both Control and CCN2-KO groups after DOX treatment. * *p* < 0.05 vs. Control. *n* = 6–10.

Regarding analysis of genes related to apoptosis, a significant increase in *Bax*, *Bcl2*, and *Mcl1* expression was found in both DOX-treated groups compared to control mice, suggesting that CCN2 does not regulate this pathway after DOX administration (Figure 6).

### 2.4. Absence of CCN2 Reduces DOX-Induced Cardiac Fibrosis

**Figure 7:**
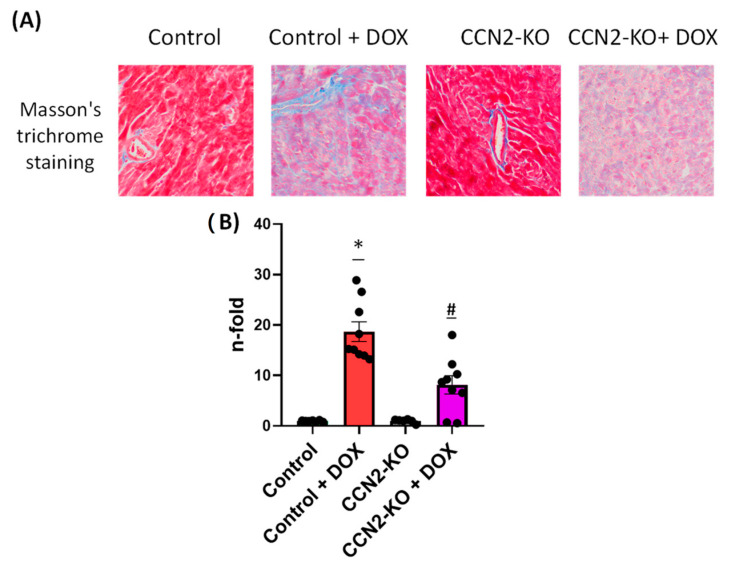
Cardiac fibrosis in mice assessed using Masson’s trichrome staining of left ventricular (LV) sections. (**A**) Representative images of the stained sections. (**B**) Quantitative analysis revealed a significant increase in fibrosis in both DOX-treated groups compared to the untreated groups. However, the Control + DOX group exhibited a greater extent of fibrosis than the CCN2-KO + DOX group. * *p* < 0.05 vs. Control; # *p* < 0.05 vs. Control + DOX. *n* = 6–10.

Cardiac fibrosis in mice was assessed using Masson’s trichrome staining of left ventricular (LV) sections (Figure 7). The analysis demonstrated a notable increase in cardiac fibrosis in mice from both DOX-treated groups compared to the untreated groups. However, the extent of fibrosis observed in the control + DOX group exceeded that in the CCN2-KO + DOX group (Figure 7), indicating that CCN2 deletion might mitigate the cardiac fibrotic effects induced by DOX administration.

## 3. Discussion

The present study aimed to investigate the potential role of CCN2 in the development of DOX-induced cardiac damage. To this end, DOX was administered to inducible CCN2-KO mice to determine if differences existed compared to mice that express CCN2. The results of this study indicate that the deletion of CCN2 mitigates several deleterious cardiac effects induced by DOX treatment, highlighting a potential protective role for CCN2 modulation in preventing DOX-associated cardiac damage. Specifically, the absence of CCN2 minimized alterations in heart function and reduced the expression of oxidative stress markers and the percentage of cardiac fibrosis caused by DOX treatment. Thus, these findings would highlight the potential protective role of CCN2 deletion against DOX-induced cardiac damage.

CCN2 is a versatile protein with complex interactions that lead to various biological effects depending on the context and environment. It plays a crucial role in embryonic development [7] and maintaining cardiovascular balance, as shown in recent studies, including ours [5,16]. Elevated levels of CCN2 are linked to several cardiovascular diseases [5]. While CCN2 deficiency is associated with increased risk of aortic aneurysm [28], its exact role in cardiac disease remains debated. Some authors, described CCN2 as an autocrine regulator of cardiac fibrosis, demonstrating that cardiomyocyte-derived CCN2 is dispensable for cardiac fibrosis, but inhibiting CCN2 induction in activated fibroblasts abrogates the cardiac fibrotic response after angiotensin II infusion [29]. Other studies indicate that conditional CCN2 deletion fails to mitigate cardiac hypertrophy and fibrosis induced by pressure overload [30]. Conversely, our present findings demonstrate a reduction in cardiac fibrosis following five days of DOX administration in the absence of CCN2. Our findings also contrast with those of another study that attributes the cardiac toxicity of the vascular endothelial growth factor receptor inhibitor sunitinib to the release of CCN2 via autophagic degradation [6]. These discrepancies may be due to differences in experimental models and the distinct mechanisms used to induce cardiac dysfunction. Altogether, this suggests a nuanced role for CCN2 in cardiac pathologies, with its effects varying significantly depending on the underlying cause of the damage.

Regarding cardiac damage induced through DOX administration in mice, several key characteristics have been described. These include a reduction in body weight after treatment [31,32,33] and a decrease in cardiac mass relative to the tibia length of the animal [34]. Consequently, in the present study, all animals treated with DOX experienced significant body weight loss, both in the control group and the CCN2-KO group. Additionally, a decrease in the heart weight/tibia length ratio was observed five days post-DOX administration in both control and CCN2-KO mice. These data suggest that CCN2 does not influence DOX-induced body and cardiac mass loss. Another reported effect in DOX-treated mice is cardiac dysfunction, characterized by a decrease in EF and FS percentages [34,35]. The findings of our study revealed that, despite a decline in cardiac mass, the CCN2-KO group subjected to DOX treatment did not display a notable decrease in cardiac function. This stands in stark contrast to the control mice, wherein DOX administration led to a significant reduction in these metrics. Hence, our results imply that CCN2 might be playing a pivotal role in the development of DOX-induced cardiac dysfunction, independent of its effect on cardiac mass.

Regarding the classic markers of DOX-induced cardiac damage, several studies have demonstrated alterations, including changes in the gene expression of cardiac α-myosin heavy chain (*Myh6*) and β-myosin heavy chain (*Myh7*). Typically, under physiological conditions, the expression of *Myh6*, the predominant isoform, decreases, while the expression of *Myh7*, characteristic of pathological conditions, increases [34]. In this study, we observed that *Ccn2* deletion did not reduce the increase in *Myh7* expression induced by DOX treatment. However, it did prevent the decrease in *Myh6* levels, which may contribute, to some extent, to the observed improvements in cardiac function.

Various studies have highlighted several molecular mechanisms involved in DOX-induced cardiac damage, including oxidative stress, apoptosis, inflammatory responses, and calcium metabolism. However, according to findings from a study [36], it seems that CCN2 may not play a significant role in the apoptotic cardiac response triggered by DOX administration. This inference is drawn from the lack of significant differences observed in the expression of pro-apoptotic (*Mcl1* and *Bax*) or anti-apoptotic (*Bcl2*) genes between mice treated with DOX alone versus those treated with DOX in conjunction with CCN2. Contrastingly, our study’s data indicate that the absence of CCN2 leads to a reduction in the expression of *Nfe2l2* (encoding NRF2) and *Hmox1*, which are commonly utilized as biomarkers of oxidative stress. In control mice, the expression of both genes significantly increases following DOX administration. These findings suggest that CCN2 might be involved in inducing oxidative stress associated with DOX-induced cardiac damage after five days of administration. Furthermore, at the protein level, an increase in NRF2 activation was observed solely in the control group treated with DOX compared to other groups. This observation implies that the mechanisms responsible for activating NRF2, leading to its transcription and subsequent translation, are diminished in the absence of CCN2 when induced by DOX. These outcomes align with previous research demonstrating that CCN2 enhances superoxide anion production in the murine aorta [37]. Similarly, a recent study demonstrated that *Hmox1* deletion prevents DOX-induced cardiomyocyte injury by downregulating CCN2 [28]. Altogether highlighted CCN2 as a potential mediator of cardiac stress oxidative damage induced by DOX administration.

Overall, the results obtained in this study indicate that CCN2 is involved in the development and progression of DOX-induced cardiac damage in mice. Under the conditions in which the study was conducted, evidence was found that the genetic deletion of CCN2 attenuates some of the negative effects associated with DOX treatment. This could be due to CCN2’s role in regulating mechanisms related to fibrosis development and oxidative stress, which could be mediating the preservation of cardiac function observed in CCN2-KO mice on the fifth day after DOX administration. In this context, previous studies have demonstrated that CCN2 is an autocrine regulator of cardiac and vascular fibrosis [7,29] as well as an inductor of oxidative stress in aorta and kidney in mice [37,38]. However, further research is necessary to precisely understand the molecular mechanisms through which CCN2 may be exerting these effects. Thus, current investigations are underway to explore the potential of CCN2 as a therapeutic target in various pathologies. In this context, an anti-CCN2 antibody (FG-3019) is being tested in several clinical trials related to diseases with elevated CCN2 levels, such as Duchenne muscular dystrophy (NCT02606136), pancreatic adenocarcinoma (NCT04229004), and pulmonary fibrosis (NCT03955146), all of which are associated with fibrotic processes.

CCN2 may exert different functions depending on the cardiac cell type. In cardiomyocytes, CCN2 promotes hypertrophy and fibrosis, contributing to adverse cardiac remodeling and dysfunction [39]. In cardiac fibroblasts, CCN2 drives extracellular matrix production, leading to increased fibrosis and tissue stiffness, which further impairs cardiac function [29]. Additionally, CCN2 influences endothelial cells by modulating angiogenesis, with context-dependent effects that can either support or exacerbate pathological outcomes [40]. The observed reduction in cardiac fibrosis and oxidative stress markers in CCN2-KO mice following DOX treatment underscores the functional implications of CCN2 in promoting DOX-induced cardiac damage. These findings suggest that targeting CCN2 could be a promising therapeutic strategy to mitigate fibrosis, oxidative stress, and the associated adverse cardiac remodeling in patients undergoing chemotherapy.

While our study presents promising findings, it is crucial to acknowledge the limitations inherent in this preclinical research based on animal models. The study was conducted with a unisystemic focus, primarily examining cardiovascular effects, which may not capture the full spectrum of mechanisms involved in cardiac dysfunction.

Furthermore, we recognize age and sex bias in our study, which may impair, at least in part, the generalizability of our findings. However, the inclusion of female mice in cardiac damage models can introduce significant variability due to hormonal influences, particularly from estrogen, which has cardioprotective effects that can alter the extent of injury, gene expression, and inflammatory responses, potentially obscuring or exaggerating experimental results [41]. Future studies should address these factors by including diverse age groups and both sexes.

Finally, age and environmental factors such as diet, physical activity, and housing conditions, which were not extensively controlled, may have impacted the results and should be standardized or more thoroughly examined in future research.

The findings of this study highlight the potential of CCN2 modulation as a promising therapeutic strategy for managing doxorubicin (DOX)-induced cardiac damage. The observed reduction in cardiac fibrosis and oxidative stress markers in CCN2-KO mice suggests that targeting CCN2 could mitigate some of the adverse effects associated with DOX treatment. Clinically, this points to the potential benefit of developing CCN2-targeted therapies or interventions to protect against chemotherapy-induced cardiotoxicity. However, given the complexity of CCN2’s role in cardiovascular health and the observed variability in its effects, further research is needed to confirm these results in human models and to clarify the precise mechanisms by which CCN2 modulates cardiac function. Additionally, as CCN2 is involved in various pathologies with elevated fibrotic processes, including Duchenne muscular dystrophy, pancreatic adenocarcinoma, and pulmonary fibrosis, ongoing clinical trials of anti-CCN2 therapies may provide further insights into its therapeutic potential across different conditions. Understanding the full spectrum of CCN2’s effects and refining strategies for its modulation could lead to more effective treatments for patients suffering from both DOX-induced cardiac damage and other CCN2-related diseases.

## 4. Materials and Methods

### 4.1. Study Approval

Animal studies were conducted at the Instituto de Investigación Sanitaria Fundación Jiménez Díaz (IIS-FJD) in compliance with current European legislation (Directive 2010/63/EU) and National regulations (Real Decreto 53/2013) for the care and use of laboratory animals, as well as following the ARRIVE Guidelines. The study protocol received approval from the IIS-FJD Animal Research Ethical Committee and the Comunidad de Madrid Committee (PROEX 065/18 and PROEX 242.2/21).

### 4.2. Generation of Tamoxifen-Inducible CCN2 Full-KO Mice and DOX Administration

Time-inducible male conditional knockout (KO) mice for the *Ccn2* gene (C57BL6/6J; CCN2flox/floxROSA26-ERT/Cre) were used. The previously described protocol has been followed [5]. Briefly, once mice were 13–14 weeks old, they were randomly divided in two groups: (1) CCN2-KO group: received intraperitoneal (IP) injections of 0.1 mL tamoxifen (10 mg/mL dissolved in corn oil; C8267, Sigma-Aldrich, St. Louis, MO, USA) four times on alternate days, and (2) Control group: received IP injections of 0.1 mL of the tamoxifen solvent (corn oil). Following a two-week washout period, *Ccn2* deletion was verified by polymerase chain reaction (PCR) using specific primers (Eurofins): Ccn2-floxed Forward: 5′-AATACCAATGCACTTGCCTGGATGG-3′ and Ccn2-floxed Reverse: 5′-GAAACAGCAATTACTACAACGGGAGTGG-3′. The PCR products were separated by size on a 1.5% agarose gel. Both CCN2-KO and control mice were divided into two groups: one receiving DOX treatment and the other receiving a vehicle solution (sodium chloride) (*n*= 6–10 per group). Body weight was recorded in every animal prior to treatment. A single intraperitoneal dose of DOX (Abcam; 15 mg/kg) was administered to some of the CCN2-KO mice (CCN2-KO + DOX) and Control mice (Control + DOX). The remaining two groups (CCN2-KO and Control) were administered an equivalent volume of the vehicle solution. The sample size per group was Control: *n* = 9; CCN2-KO: *n* = 6; Control + DOX: *n* = 10; CCN2-KO + DOX: *n* = 10), as specified in approved version by Animal Research Ethical Committee.

**Figure 8:**
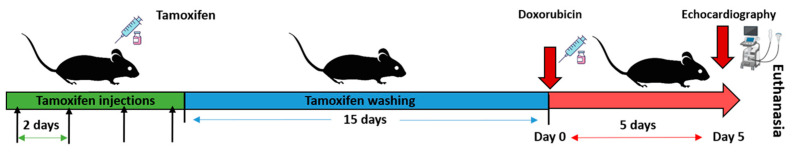
Schematic representation of the experimental model. The diagram outlines the various treatment groups and experimental procedures used in the study, illustrating the administration of DOX and CCN2 deletion applied to the mice.

Five days post-treatment, body weight was measured again, and echocardiograms were conducted. Mice were then euthanized using an overdose of isoflurane (Abbott Laboratories, Chicago, IL, USA). Immediately, the hearts were excised, weighed, and this measure was normalized to the corresponding tibia length. The heart samples were processed considering subsequent procedures. The hearts were transversely sectioned, separating the pole regions, both apex and atria, quickly frozen in liquid nitrogen, and stored at −80 °C for gene expression studies. The remaining tissue was fixed in 4% paraformaldehyde (PFA) at pH 7.4 and embedded in paraffin for histological studies (Figure 8).

### 4.3. Evaluation of Cardiac Function by Echocardiography

Cardiac studies were performed using a portable LoGIQ-e ultrasound system equipped with a GE L10-22 MHz ultrasound probe (GE-Healthcare, Arlington Heights, IL, USA). Mice were anesthetized using 3% isoflurane and 1 L/min 100% oxygen in a chamber once animals lost righting reflex. After that, mice were laid supine on a heated surgical platform (SurgiSuite, Kent Scientific Corporation, Torrington, CT, USA) and anesthetized by isoflurane (2%) administered by a nosecone. Although all procedures were adapted to standardize a range of heart rates adequate to perform the measurement (400–500 bpm) according to previous studies [42], it was impossible to measure this parameter because the apparatus could not accurately obtain the heart rate. Left ventricle (LV) structural and functional parameters were derived from the parasternal short-axis view. A perpendicular M-mode cursor was positioned across the midpoint of the LV anterior and posterior walls to measure chamber dimensions. LV internal diameter (LVID) was quantified at the end-diastole (LVIDd) and end-systole (LVIDs). Data were averaged from measurements of at least three cardiac cycles. Ejection fraction (EF) and fractional shortening (FS) were calculated using the following formulas: % EF = ((LVIDd^3^ − LVIDs^3^)/LVIDd^3^) × 100 and % FS = ((LVIDd − LVIDs)/LVIDd) × 100, as previous described [43]. All measurements were performed in a blinded manner by two independent observers.

### 4.4. Gene Expression Studies by RT-qPCR

Cardiac messenger RNA (mRNA) was extracted using TRIzol method, as previously described [37]. The extracted mRNA was quantified using a NanoDrop spectrophotometer (Thermo Scientific, Waltham, MA, USA). Complementary DNA (cDNA) synthesis was performed using 2 µg of mRNA and the High-Capacity cDNA Reverse Transcription Kit (Applied Biosystems, Foster City, CA, USA) according to the manufacturer’s guidelines. Gene expression analysis was conducted using RT-qPCR on a Real-Time FAST PCR 7500 system (Life Technologies, Carlsbad, CA, USA), the Premix Ex Taq (Probe qPCR) Master Mix (TakaRa, Kyoto, Japan), and pre-designed oligonucleotides from Integrated DNA Technologies (IDT). The reaction cycle conditions were as follows: 1 cycle of 2 min at 50 °C, 1 cycle of 10 min at 95 °C, and 45 cycles of 15 s at 95 °C followed by 60 s at 60 °C. Expression levels were normalized to the Glyceraldehyde 3-phosphate dehydrogenase (GAPDH) gene (IDT), and relative expression was calculated using the 2^−ΔΔCt^ method.

### 4.5. Detection of NRF2 Activation by Immunohistochemistry

To investigate NRF2 protein activation in the heart, 2 µm sections of the left ventricles (LVs) underwent a meticulous preparation process. This involved overnight incubation at 65 °C, deparaffinization in xylene, hydration in a decreasing ethanol gradient, and antigen retrieval using a PT-Link system with EnVision FLEX solution at pH 9. To block endogenous peroxidase activity, tissues were treated with 3% H_2_O_2_ followed by a secondary blocking step using a diluted casein solution. After incubation with a primary antibody against phosphorylated NRF2 protein (Abcam, Waltham, MA, USA) overnight at 4 °C, sections were washed and incubated with a secondary antibody. Detection was achieved using diaminobenzidine solution, followed by counterstaining with hematoxylin. Tissue sections were dehydrated, cleared, and mounted using DPX (Sigma-Aldrich) before imaging with a DMD108 microscope (Leica Microsystems, L’Hospitalet de Llobregat, Spain) and analysis using Image Pro Plus 6.0 software (Media Cybernetics, Rockville, MD, USA).

### 4.6. Evaluation of Cardiac Fibrosis

Cardiac fibrosis was assessed through the detection of collagen fibers using Masson’s trichrome staining. For this purpose, 2 µm sections of the LVs from mice hearts were prepared, and the staining was conducted utilizing a Masson Trichrome staining kit (Bio-Optica, Milan, Italy) according to the manufacturer’s protocol. Post-staining, images were acquired with the Leica DMD108 microscope and analyzed with Image-Pro-Plus software 6.0.

### 4.7. Statistical Analysis

Data are expressed as the mean value ± standard error of the mean (SEM). Normality of the data was assessed using the Shapiro–Wilk test. To compare a variable across multiple groups, one-way ANOVA or Kruskal–Wallis tests were used, followed by Tukey’s or Dunn’s post hoc tests, depending on the normality. Statistical analyses were performed using GraphPad Prism 8.0 software (GraphPad Software, San Diego, CA, USA). Differences between groups were considered statistically significant at *p* < 0.05.

## Data Availability

The data supporting the results of this study are available from the corresponding authors upon reasonable request.

## Supplementary Materials

The following supporting information can be downloaded at: https://www.mdpi.com/article/10.3390/ijms25179617/s1.
