# Supplementary material for: Ccn2 Deletion Reduces Cardiac Dysfunction, Oxidative Markers, and Fibrosis Induced by Doxorubicin Administration in Mice
Source: Int J Mol Sci. 2024 Sep 5;25(17):9617. doi: 10.3390/ijms25179617 (PMC11394698; doi:10.3390/ijms25179617)
Supplement: Supplementary file 1 [file ijms-25-09617-s001.zip › ijms-3137840-supplementary.pdf]

Supplementary Figure S1

% Ejection fraction

% EF = ((LVIDd³ – LVIDs³) / LVIDd³) x 100

Control	Control + DOX	CCN2-KO	CCN2-KO + DOX
68,5609777	61,4937378	68,7393739	77,0123049
66,8183887	45,9580175	66,6351292	69,7468654
72,9986144	61,9172107	74,9035958	61,8921875
69,6704865	65,1870728	71,7376709	74,6730613
66,5102023	62,4569842	67,9690917	58,8850115
74,2299023	61,25489315	66,6706963	69,25364812
68,254125	60,1235467		60,2145236
73,1546827	55,25687451		61,21356489
71,2564852	61,32185467		72,21456981
	60,25364854		65,21458745

% Fractional shortening

% FS = ((LVIDd – LVIDs) / LVIDd) x 100

Control	Control + DOX	CCN2-KO	CCN2-KO + DOX
32,0056899	27,2604588	32,3412698	38,7429644
30,7692308	18,5714286	30,6543385	32,8947368
35,3658537	27,5630252	36,9230769	27,5128451
32,8125	29,6875	34,375	36,7647059
30,5555556	28,12458662	31,5789474	25,6410256
36,3636364	24,12541256	30,6666667	33,02154785
31,1524365	26,21547856		30,21475691
32,2546597	29,2154687		28,12548545
33,2153647	27,12532145		27,93365215
	26,12548841		29,21455671

Note: Due to technical limitations, values for RVID, LV trace, and LA could not be obtained for the mice.

<u>Supplementary Figure S2</u>

Control

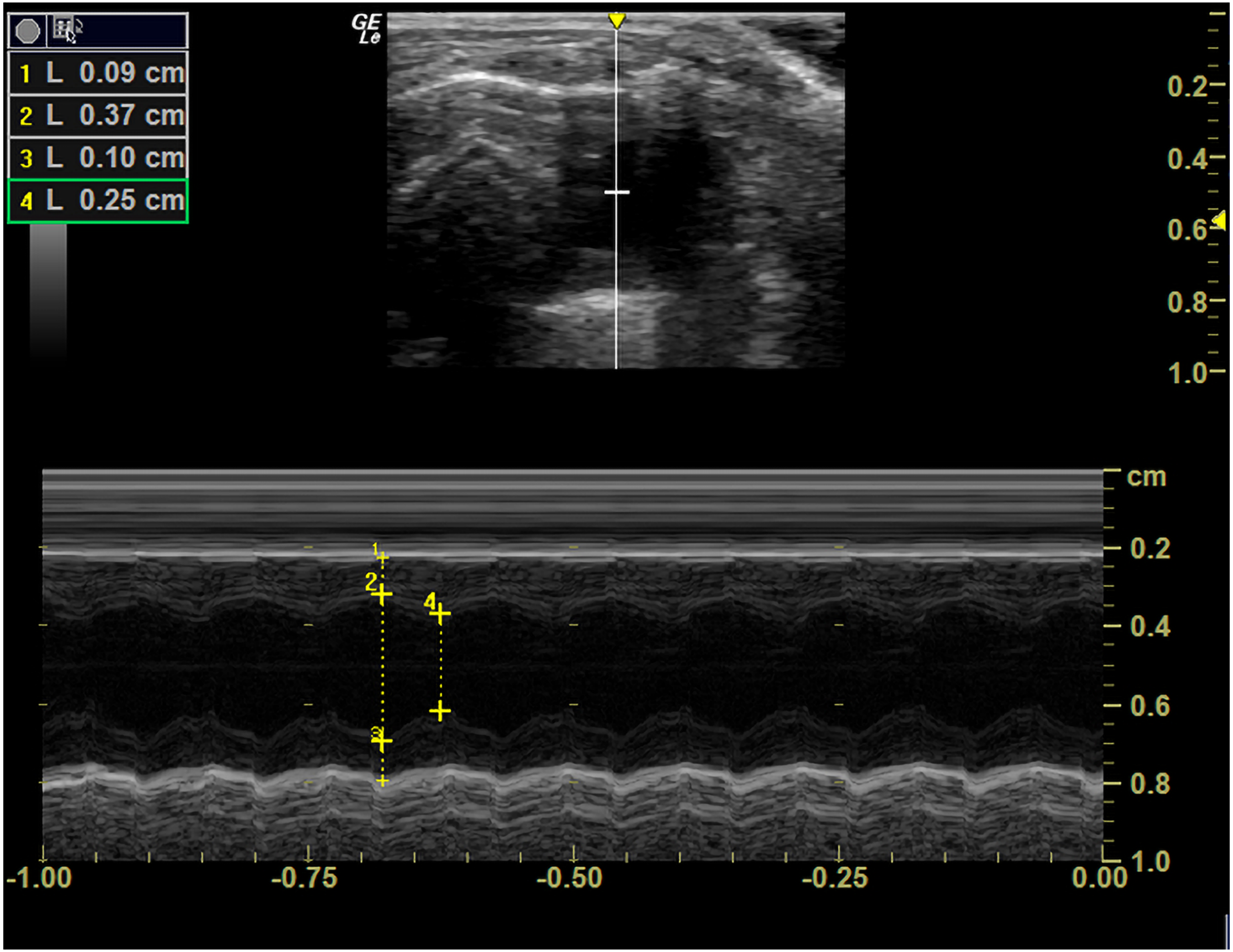

Control + DOX

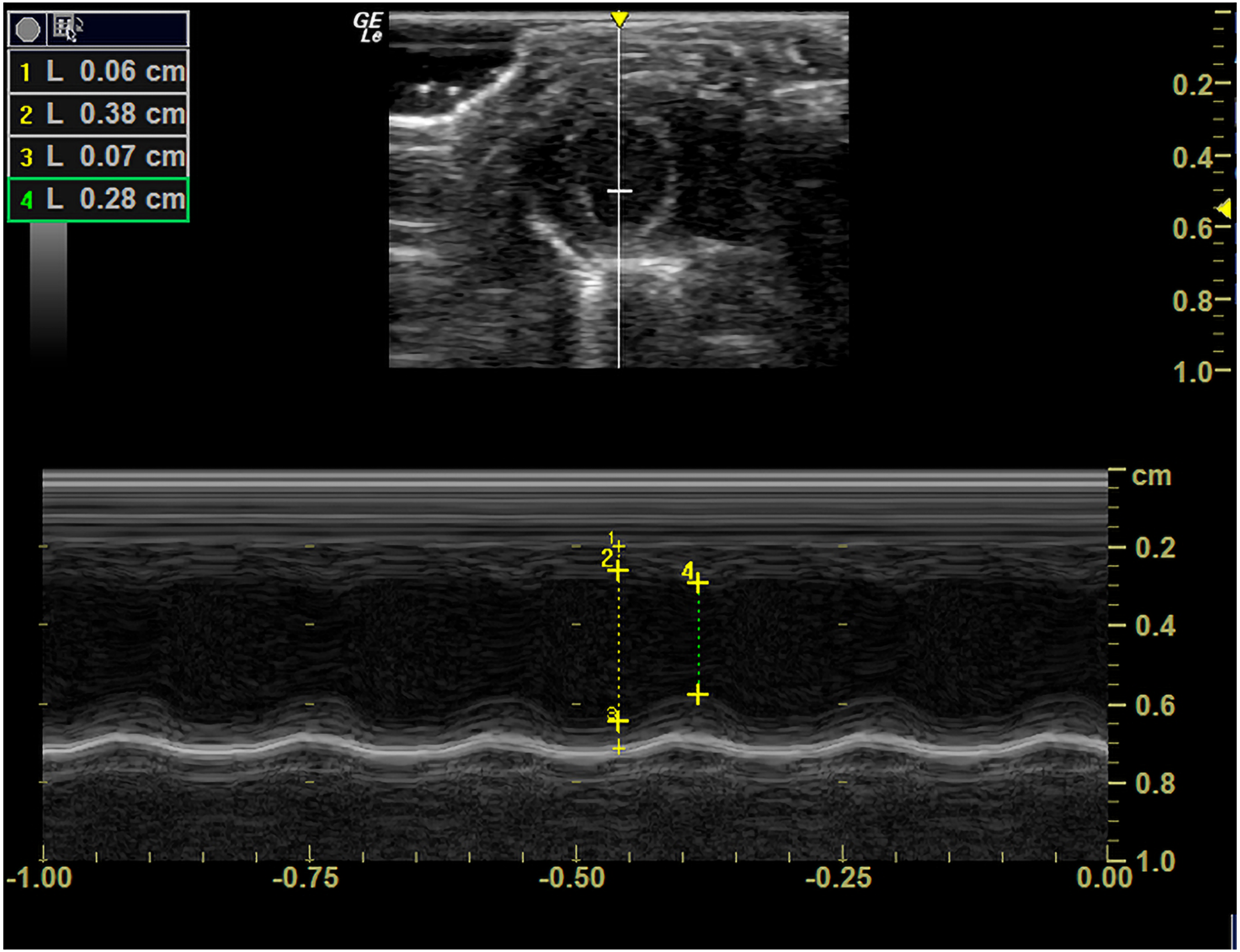

1 L 0.06 cm
2 L 0.38 cm
3 L 0.07 cm
4 L 0.28 cm
GE
0.2
0.4
0.6
0.8
1.0
cm
0.2
0.4
0.6
0.8
1.0
-1.00
-0.75
-0.50
-0.25
0.00

CCN2-KO

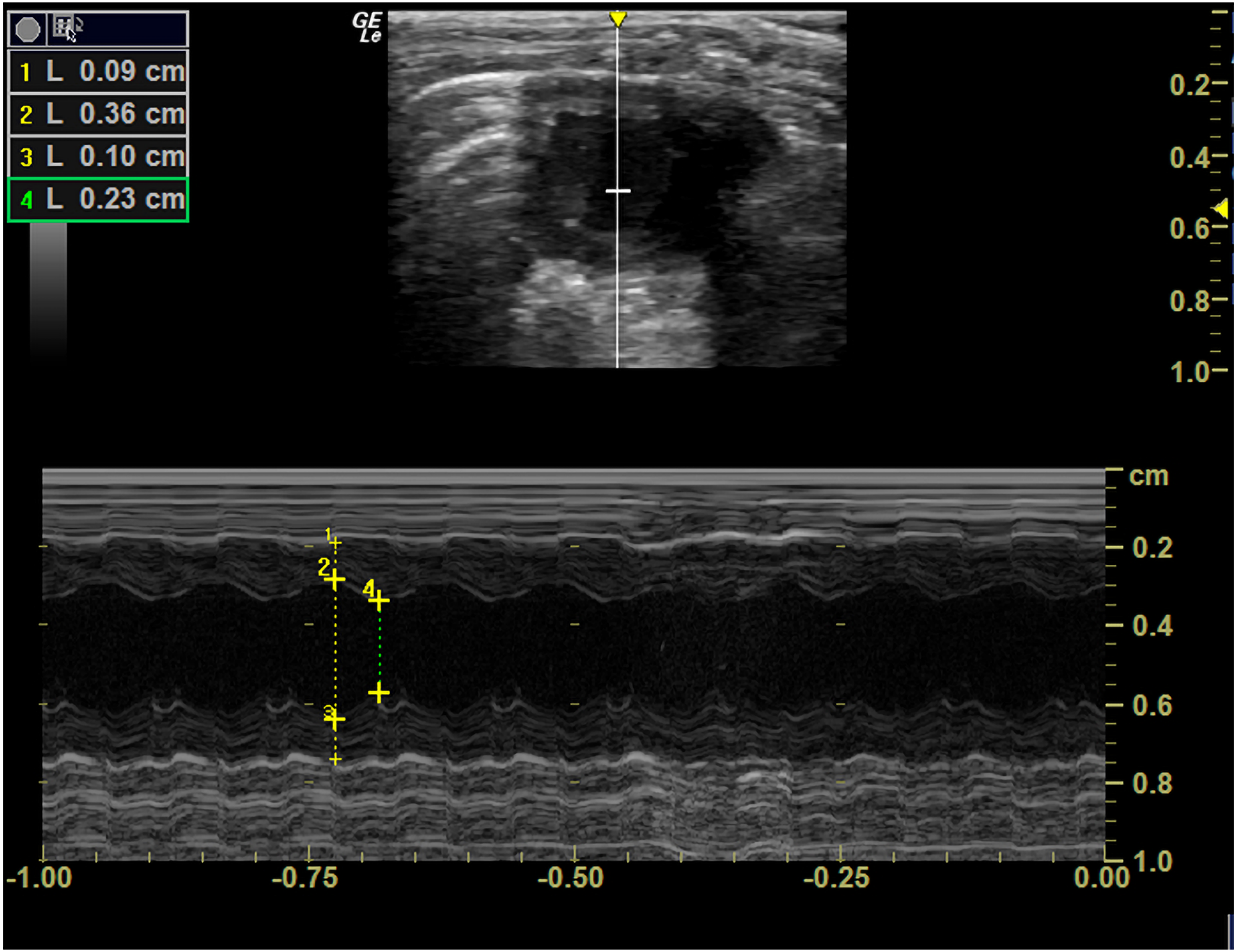
1 L 0.09 cm
2 L 0.36 cm
3 L 0.10 cm
4 L 0.23 cm
GE
Le
0.2
0.4
0.6
0.8
1.0
cm
0.2
0.4
0.6
0.8
1.0
-1.00
-0.75
-0.50
-0.25
0.00

CCN2-KO + DOX

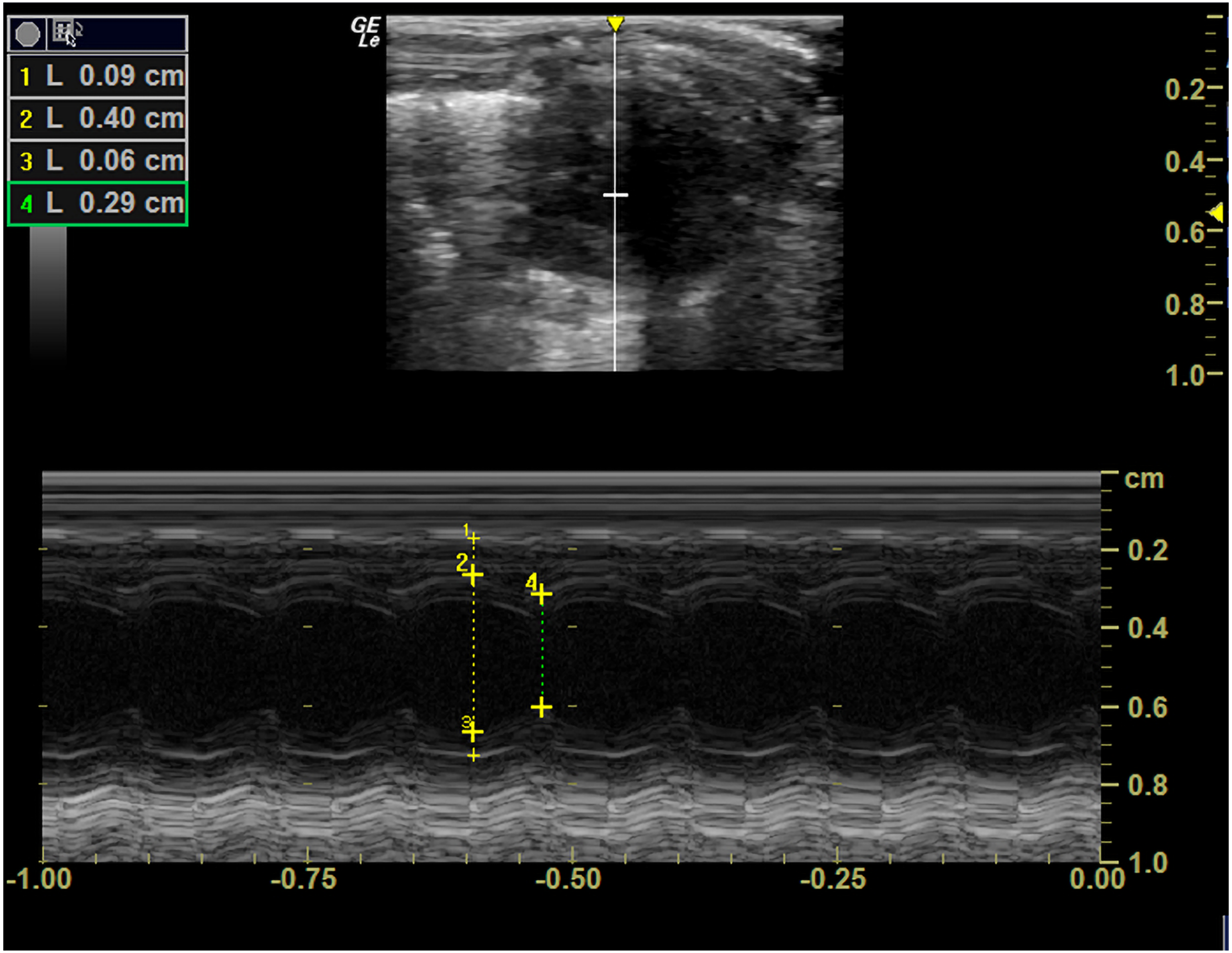

1 L 0.09 cm
2 L 0.40 cm
3 L 0.06 cm
4 L 0.29 cm
0.2
0.4
0.6
0.8
1.0
cm
0.2
0.4
0.6
0.8
1.0
-1.00
-0.75
-0.50
-0.25
0.00
